# Supplementary material for: Entanglement in photoionisation reveals the effect of ionic coupling in attosecond time delays
Source: Nat Commun. 2025 Sep 29;16:8554. doi: 10.1038/s41467-025-64182-8 (PMC12480667; doi:10.1038/s41467-025-64182-8)
Supplement: Supplementary file 1 — Supplementary Information [file 41467_2025_64182_MOESM1_ESM.pdf]

Supplementary Information
Entanglement in photoionisation reveals the effect of ionic coupling in attosecond time delays

Ioannis Makos[1], David Busto[1,2], Jakub Benda[3], Dominik Ertel[1], Barbara Merzuk[1], Benjamin Steiner[1], Fabio Frassetto[4], Luca Poletto[4], Claus Dieter Schröter[5], Thomas Pfeifer[5], Robert Moshammer[5], Serguei Patchkovskii[6], Zdeněk Mašín[3], and Giuseppe Sansone[1,7]

[1]Institute of Physics, University of Freiburg, Hermann-Herder-Straße 3, 79104 Freiburg, Germany
[2]Department of Physics, Lund University, PO Box 118, SE-221 00 Lund, Sweden
[3]Institute of Theoretical Physics, Faculty of Mathematics and Physics, Charles University, V Holešovičkách 2, 180 00, Prague 8, Czech Republic
[4]Istituto di Fotonica e Nanotecnologie, CNR, 35131 Padova, Italy
[5]Max-Planck-Institut für Kernphysik, 69117 Heidelberg, Germany
[6]Max Born Institute, Max-Born-Str. 2A, D-12489 Berlin, Germany
[7]Freiburg Institute for Advanced Studies (FRIAS), University of Freiburg, Albertstraße 19, 79104 Freiburg, Germany

1.1 Supplementary information on experimental setup

Extreme ultraviolet (XUV) spectra consisting of odd harmonics of the driving infrared (IR) field have been produced in krypton. A typical XUV spectrum is shown in Supplementary Fig. 1. The spectra were used to ionise a mixture of CO_2 molecules and argon atoms (CO_2-Ar concentration of 2:1) to obtain XUV-only and two-colour photoionisation traces obtained under the same experimental conditions. The typical time-of-flight mass spectrum obtained by single XUV photoionisation (measurements without IR field) is shown in Supplementary Fig. 2. The TOF shows the presence of the parent molecular ion (CO_2^+) and the singly ionised argon Ar^+. In addition, photoionisation from the neutral molecule can lead to fragmentation into additional channels including CO^+ and also O^+.

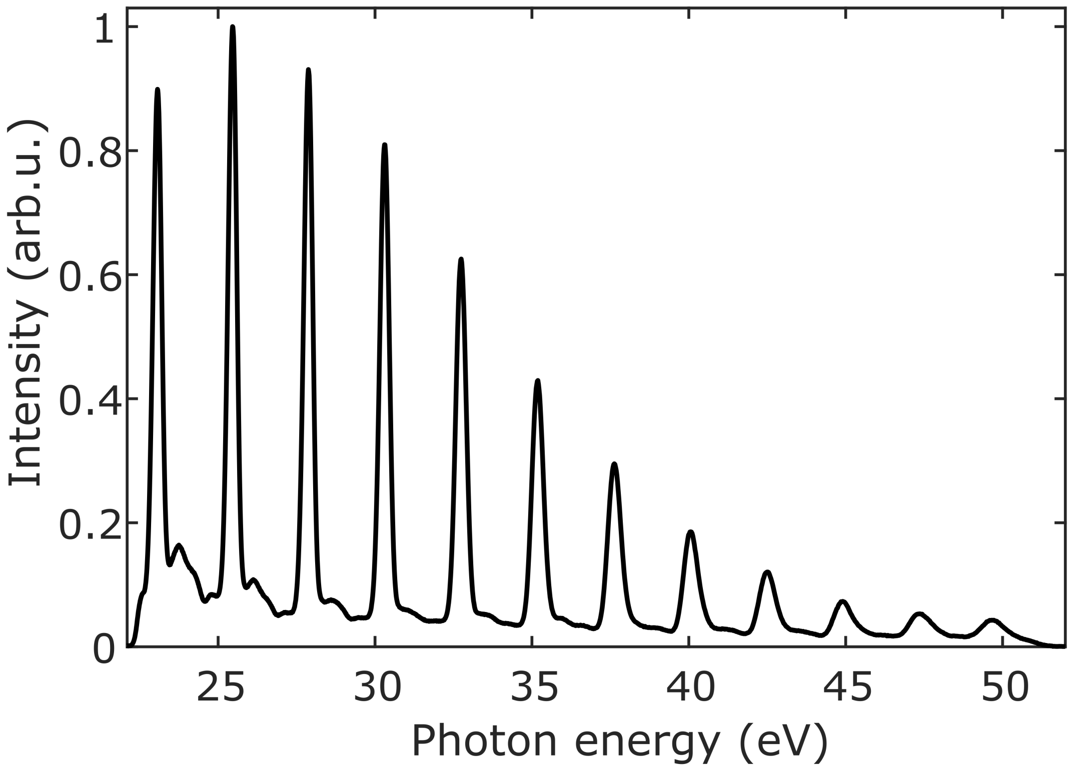

Supplementary Fig. 1: **XUV spectrum.** Typical XUV spectrum generated in krypton.

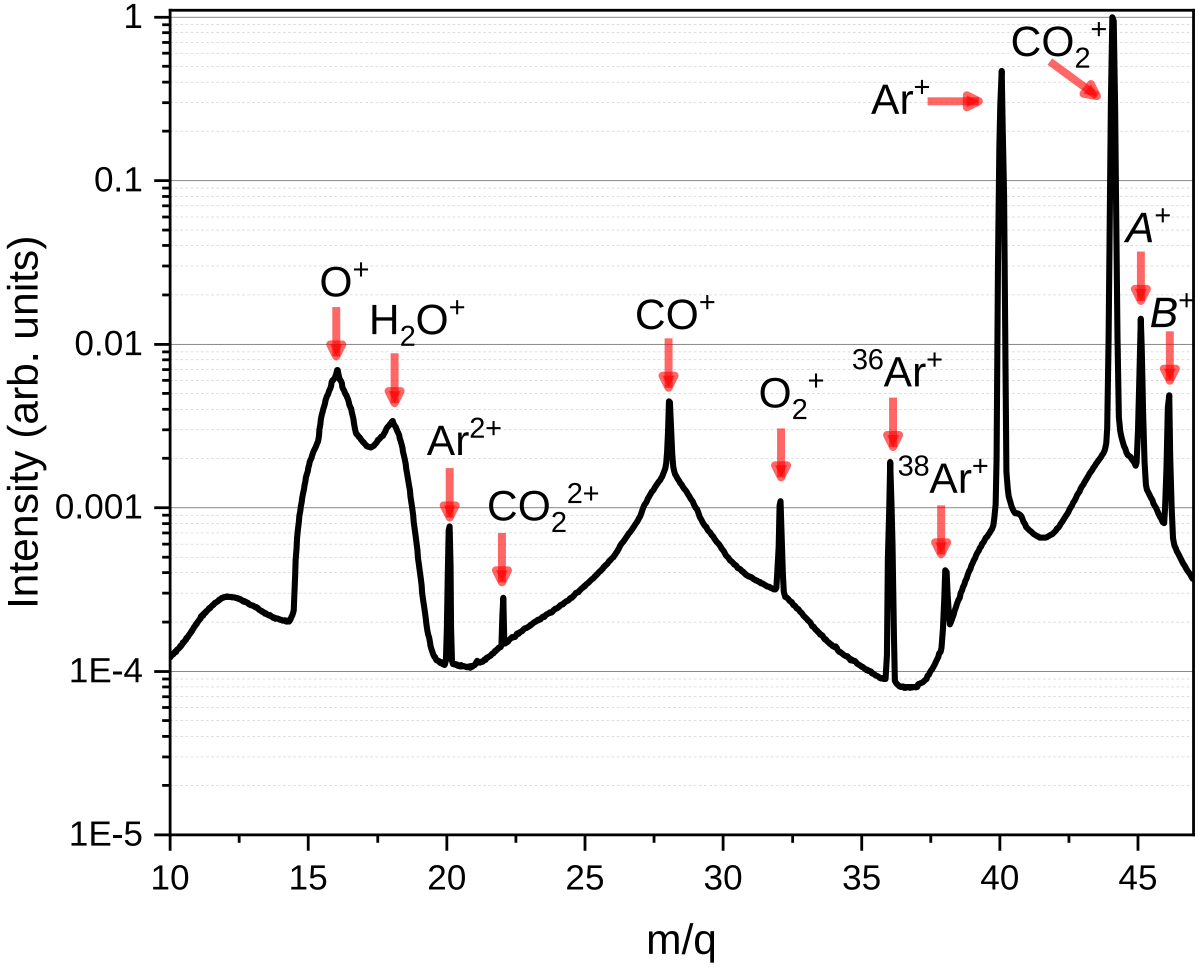

Supplementary Fig. 2: **Time-of-flight mass spectra acquired in the CO_2-Ar mixture.** TOF spectra (m/q) ratio measured in the CO_2-Ar mixture. $A^+ \equiv {}^{13}CO_2^+$; $B^+ \equiv (CO^{18}O)^+$

1.2 Supplementary information on data analysis

Photoelectron spectra are selected in coincidence with the ionic fragments CO_2^+ and Ar^+. The phase of the sideband oscillations were extracted by using a Fourier Transform algorithm and by monitoring the peak corresponding to the 2ω oscillations in the Fourier spectrum. For the measurements presented in the main manuscript in Fig. 3, the variation of the phase as a function of the photoelectron energy is reported in Supplementary Fig. 3. It can be observed that, around the region in which we expect to observe the sideband related to initial photoionisation from the $B^2\Sigma_u^+$ state, the phase of the 2ω component is rather flat, indicating that the contribution of the sidebands from the $B^2\Sigma_u^+$-state can be isolated by considering the average value around a small energy-region (typically $\approx$300 meV).

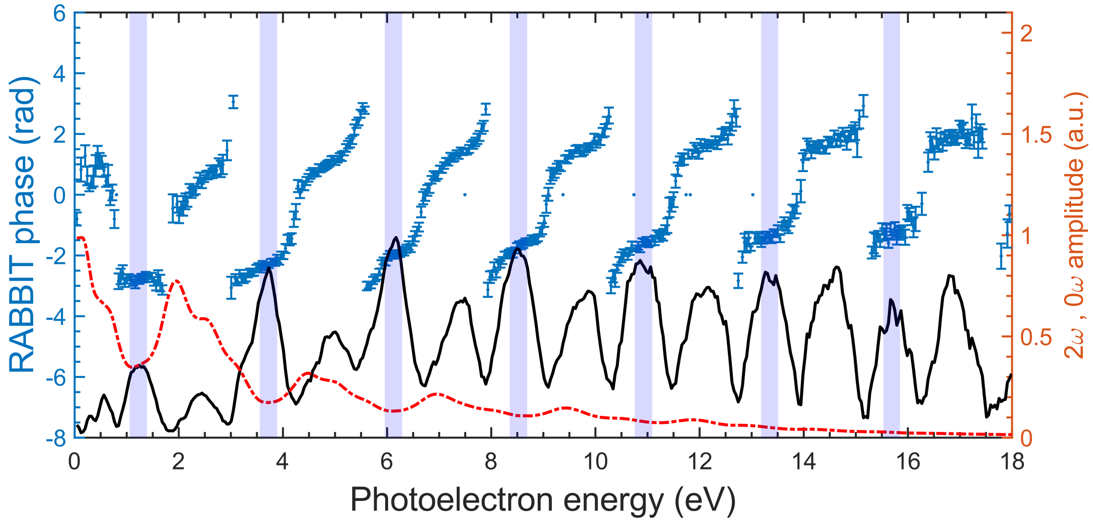

Supplementary Fig. 3: **Amplitude and phase of the RABBIT oscillations**. Energy-resolved amplitudes A_0 (red dashed line), A_2 (black line) and phase $\Delta\varphi_{atto} + \Delta\varphi_{mol.}$ (blue points) (see Eq. 1) of the photoelectron spectra associated to the CO_2^+ ion. The photoelectron spectra were integrated over the angle interval $(0° - 90°)$. The blue shaded areas indicate the energy range of the sidebands associated to the $B^2\Sigma_u^+$ cationic states. The error bars (one standard deviation) were derived from fitting the periodic oscillations of the photoelectron spectra using Eq. 1 for each photoelectron energy interval.

The RABBIT trace measured in coincidence with the Ar^+ ion is shown in Supplementary Fig. 4. The phase of the sideband oscillations was extracted using the procedure described above and is shown in Supplementary Fig. 5 for different integration angles along the common polarisation direction of the XUV and IR fields. The evolution of the phase as a function of the sideband order is due to the attochirp. The extracted phase does not significantly depend on the integration angle.

The photoionisation time delays $\tau = \tau_B - \tau_{\mathrm{Ar}}$ were obtained as the difference of the delays derived from RABBIT traces measured in coincidence with CO_2^+ and Ar^+. The comparison between the experimental time delays τ and those obtained from the full model and the model without B-C coupling for RABBIT traces integrated over the photoelectron emission angle $(0° - 20°)$ and $(0° - 50°)$ are presented in Fig. Supplementary Fig. 6a and b, respectively. For the experimental points at 28.98 eV and 31.42 eV, the deviation from the model without B-C coupling is 2.75σ and 2.47σ, 2.57σ and 2.68σ, and 1.83σ and 1.57σ for the integration angles $0° - 20°$, $0° - 50°$, and $0° - 90°$, respectively (where σ indicates the corresponding standard deviation for each experimental point).

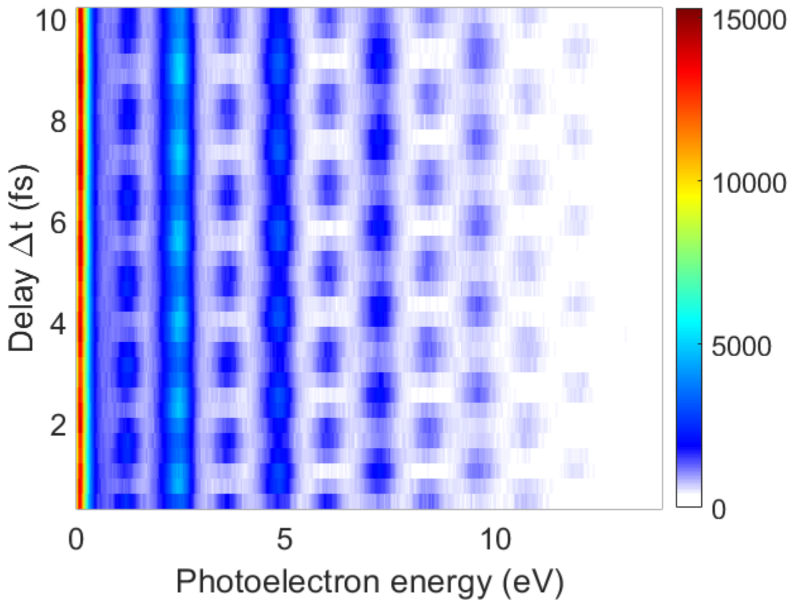

Supplementary Fig. 4: **RABBIT trace in argon.** RABBIT trace measured in coincidence with the argon ions and integrated over the angle interval $(0^\circ - 90^\circ)$.

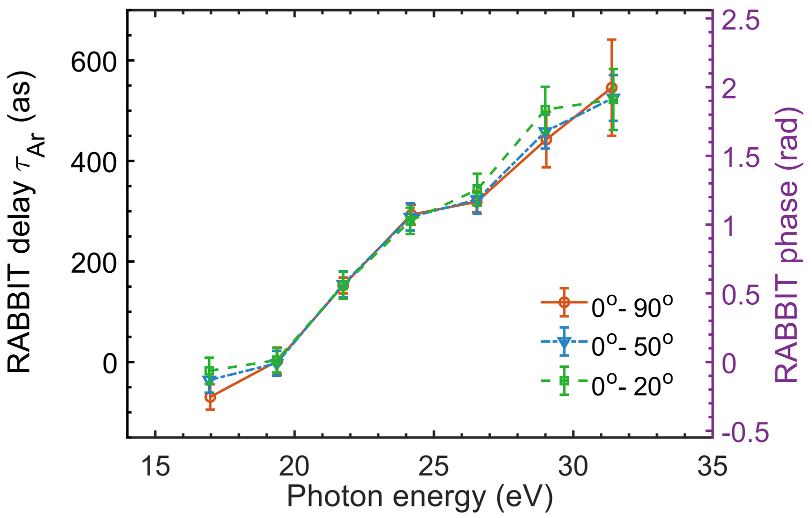

Supplementary Fig. 5: **Experimental photoionisation time delays in argon.** Photoionisation time delays measured in argon considering the photoelectrons emitted within three angle intervals: $(0° - 90°)$ (orange circles), $(0° - 50°)$ (blue triangles), $(0° - 20°)$ (green squares). The time delays were shifted by the same interval as in Fig. 2d. The error bars (one standard deviation) were derived from fitting the periodic oscillations of the photoelectron spectra using Eq. 1.

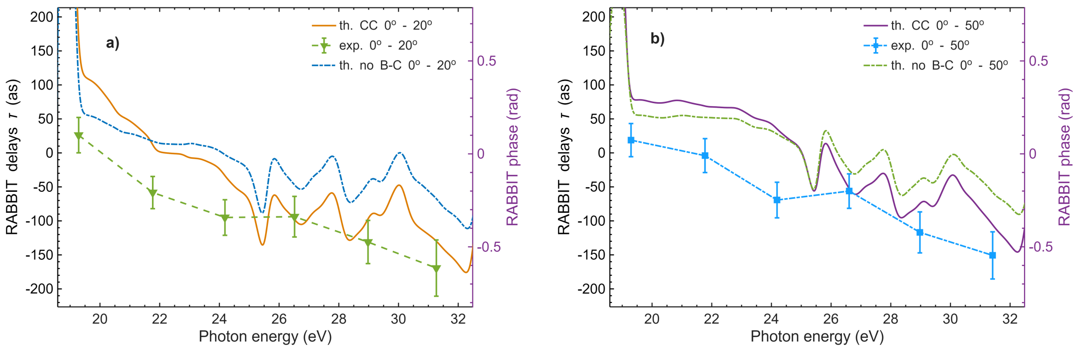

Supplementary Fig. 6: **Comparison experiment-theory of the photoionisation time delays for the integration angles** $0° - 20°$ **and** $0° - 50°$**.** (a) Experimental (green triangles (a) and blue squares (b)) difference of the photoionisation time delays $\tau = \tau_B - \tau_{\mathrm{Ar}}$ measured in coincidence with CO_2^+ and Ar^+ integrated over the photoelectron emission angle $(0° - 20°)$ (a) and $(0° - 50°)$ (b). The error bars (one standard deviation) were derived from fitting the periodic oscillations of the photoelectron spectra using Eq. 1. Same difference of the photoionisation time delays τ extracted from the theoretical models using the full model without coupling in the ionic states (blue (a) and green (b) dashed-dotted line) and the full model including the ionic coupling (yellow (a) and purple (b) solid line).

1.3 Supplementary information on theoretical model

Role of resonances and structured continua around 24 eV

One of the reasons why the theoretical model used to interpret the present measurement does not reproduce the dip in the delays at 24.19 eV (see Fig. 3) might be an insufficient description of complex near-threshold effects in photoionisation of CO_2. The one-photon cross sections and asymmetry parameters for ionization of CO_2 to the $B^2\Sigma_u^+$ (B) and $C^2\Sigma_g^+$ (C) states contain several non-trivial features at photon energies close to the photon energy of 24 eV. In particular, Siggel et al.[1] points out the presence of a resonance in the B-state at the photon energy of around 23 eV. This resonance appears in our calculations too. Even though the structure is not very conspicuous in the cross section measurement (Supplementary Fig. 7a), it might possibly affect RABBIT delays in the B state (Supplementary Fig. 7e) at the combined XUV+IR energy of around 24 eV when coupling to the nuclear motion is included. However, our fixed-nuclei calculations are not able to ascertain this point.

Another possible explanation is provided by detailed analysis of the one-photon asymmetry parameters for photoionisation into the C state of CO_2^+. While the asymmetry parameter for the B state is smooth around the energy of 24 eV, the C state angular distributions display a double well structure in the energy region 21-31 eV, as shown in Supplementary Fig. 7d. The first well below 25 eV originates from coupling of the C state to B state via electron correlation (long-range transition dipole) while the upper well most likely stems from an autoionising resonance. Our calculations reproduce the experiment very well although the autoionising structure is shifted to higher energies by approximately 2-3 eV, which is an expected accuracy of the model.

In the process of two-photon ionisation structures appearing in the C state continuum can be transferred into the B state continuum due to the IR-induced ionic coupling. Since the interference principle of the RABBIT measurement requires equality of the photoelectron energies, we need to look at structures in one-photon data which lead to the given sideband photoelectron

energy (see Fig. 4). This is indicated in Supplementary Fig. 7 by the thick dashed lines. The thick higher-energy vertical dashed line in Supplementary Fig. 7b,d corresponds to the photoelectron continuum from the C state that could interfere with the B state continuum (in the sideband at 24 eV) as a result of the ionic IR-induced coupling. This part of the C-state spectrum is very close to the center of the double well structure in the experiment of Siggel et al.[1] which occurs at around 25 eV.

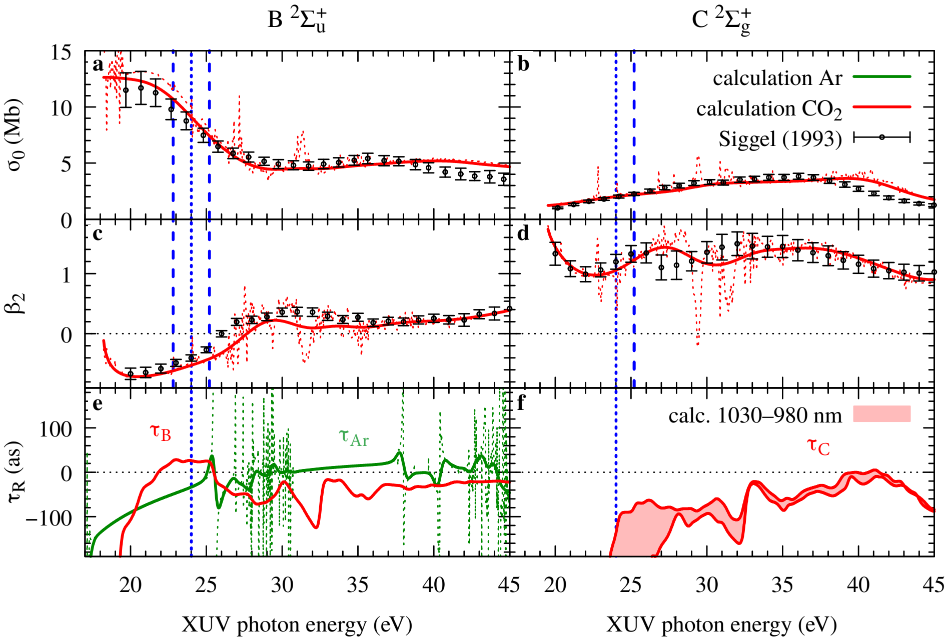

Supplementary Fig. 7: **Coupling delay for different wavelengths of the driving field.** Smoothed and non-smoothed isotropic cross section of photoionization of CO_2 to the B and C final states of CO_2^+ (panels a, b), corresponding asymmetry parameters (panels c, d), and smoothed absolute RABBIT delays τ_R ($R = B, C, Ar$) for the B-state ($\tau_R = \tau_B$) (panels e), together with both smoothed and non-smoothed calculated reference argon RABBIT delays ($\tau_R = \tau_{Ar}$) (green), obtained for 1030-nm IR. The RABBIT delays into the C-state ($\tau_R = \tau_C$) (f) are strongly sensitive to the IR wavelength, so a range is given (upper curve for 1030-nm IR; lower curve for 980-nm IR). The experimental data are from Ref. 1. The dotted vertical line marks the sideband energy of 24 eV, while the dashed vertical lines mark energies of the XUV photons (shifted by $\pm\omega_{\mathrm{IR}}$ with respect to the sideband).

In our theoretical modelling the center of the double well structure is upshifted of approximately

2-3 eV (around 27-28 eV). In the calculations, this feature is clearly coupled to a sharp drop in the delay τ_B for the B state around 25.5 eV, as shown in Supplementary Fig. 7e. Therefore we ascribe the calculated drop in the time-delay for the B state to the presence of the double well structure in the C-state spectrum. Shifting the position of this drop by 2–3 eV, to account for the correct experimental position of the autoionising resonance, would lead to qualitative better agreement with the experimental result at 24.19 eV. Furthermore, the calculated delay for the C state, see Supplementary Fig. 7f, displays a sharp jump in the region 23-27 eV, which is highly sensitive to the IR wavelength. This further supports the source of the drop in the delay in the B state continuum as a result of the coupling of the C-state continuum via the IR-induced ionic transitions. In the present calculations the drop in the CO_2 time-delay for the B state is partially cancelled by the sharp variation contributed by the autoionising resonance in Argon. Clearly, the region around 24 eV and below can be expected to be sensitive to the structured continua in the vicinity of the B and C state thresholds of CO_2.

Coupling delay

Coupling delay is a direct consequence of Rabi cycling when an effectively two-level system is driven by a laser field that is close to the resonant transition energy. After the XUV ionisation step the photoelectrons fly away from the target and they interact with the IR field, undergoing continuum-continuum transitions. In the meantime, the residual ion is also interacting with the IR field. Even though the photoelectron left the residual ion (e.g.) in state $B^2\Sigma_u^+$ (B), after some time the residual ion is in superposition of B and $C^2\Sigma_g^+$ (C) (see Supplementary Fig. 8). But we measure photoelectrons in coincidence with a given residual ion state, effectively projecting the total wavefunction on pure B or pure C. This means that what we interpret as the wave packet coupled to B is actually a mixture of wave packets coupled to B (dominant part) and to C (perturbation part). Obviously, the phase (delay) of such a combined wave packet is different

than either of the pure wave packets.

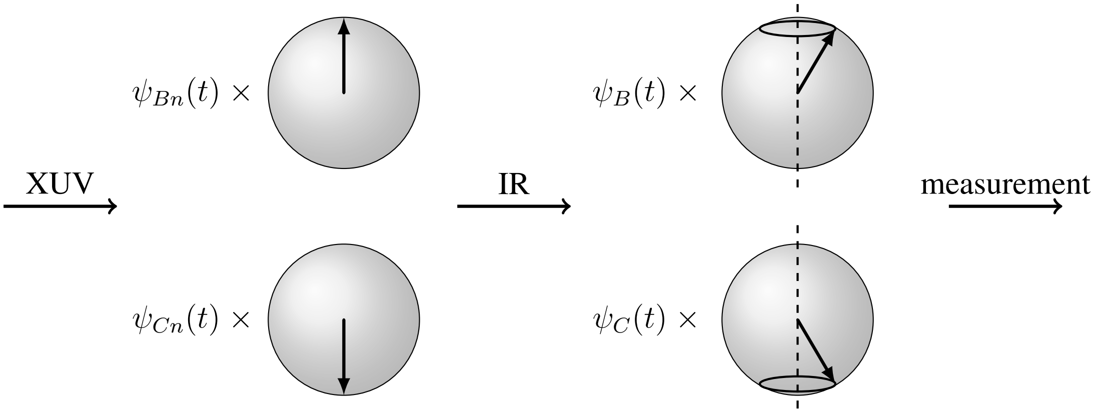

Supplementary Fig. 8: **RABBIT in dipole-coupled laser-driven system.** Schematic representation of the coupling induced by the IR field on the entangled state generated by the XUV photoionisation process. $\psi_{Bn}(t)$ ($\psi_{Cn}(t)$) indicates the time-dependent wave function of the photoelectron associated to the B (C) cationic state. $\psi_B(t)$ ($\psi_C(t)$) indicates the wave function of the photoelectron in the intial state ψ_{Bn} (ψ_{Cn}) after the interaction with the IR field. The initial state of the cation is schematically represented by vertical arrows. The interaction with the IR field leads to precession of the state vectors along the vertical directions.

Residual ion states

Already at the beginning we make several important simplifications. First, we restrict ourselves to the B and C states of the residual ion only. We let these two states become basis vectors in the space of residual ion states and represent them as two-component vectors

$$\Phi_B = \begin{pmatrix} 1 \\ 0 \end{pmatrix}, \qquad \Phi_C = \begin{pmatrix} 0 \\ 1 \end{pmatrix}. \tag{1}$$

In the time-dependent field free picture, they will have the form

$$\Phi_B^{(0)}(t) = \begin{pmatrix} 1 \\ 0 \end{pmatrix} \mathrm{e}^{-\mathrm{i}\mathcal{E}_B t}, \qquad \Phi_C^{(0)}(t) = \begin{pmatrix} 0 \\ 1 \end{pmatrix} \mathrm{e}^{-\mathrm{i}\mathcal{E}_C t}, \tag{2}$$

where $\mathcal{E}_{B,C}$ are the energies of these two states, $H_{\text{ion}}\Phi_{B,C} = \mathcal{E}_{B,C}\Phi_{B,C}$. They satisfy the time-dependent Schrödinger equation

$$\mathrm{i}\dot{\Phi}_{B,C}^{(0)}(t) = H_{\text{ion}}\Phi_{B,C}^{(0)}(t)\,. \tag{3}$$

We assume that the states follow Eq. (2) after the XUV ionisation step and that the total ("intermediate") state of the whole system (residual ion + photoelectron) can be written as superposition of one-electron wavefunction $\psi_{Bn}(t)$ coupled to B and one-electron wavefunction $\psi_{Cn}(t)$ coupled to C,

$$\Psi_n(t) = \psi_{Bn}(t)\Phi_B^{(0)}(t) + \psi_{Cn}(t)\Phi_C^{(0)}(t)\,. \tag{4}$$

We also assume a very specific form of the photoelectron wavefunctions, simply given by the stationary outgoing spherical wave solutions,

$$\psi_{Bn}(t) = \psi_{Bn}^{(+)}\mathrm{e}^{-\mathrm{i}e_{Bn}t}\,, \qquad \psi_{Cn}(t) = \psi_{Cn}^{(+)}\mathrm{e}^{-\mathrm{i}e_{Cn}t}\,, \tag{5}$$

where e_{Bn} and e_{Cn} are the ("intermediate") photoelectron momenta. Writing the photoelectron wavefunction like this assumes monochromatic waves and perfect separation of the XUV and IR steps (i.e., there is enough time for the photoelectron to develop these asymptotic solutions). In other words, it is a first step towards the standard asymptotic approximation.

The "intermediate" wavefunction (4) is the initial condition for the IR dynamics, which is the solution of the full time-dependent Schrödinger equation

$$\mathrm{i}\frac{\mathrm{d}}{\mathrm{d}t}\Psi(t) = [H_{\text{ion}} + h]\Psi(t) - \begin{pmatrix} \mathbf{r}\cdot\mathbf{F}(t) & \mathbf{D}\cdot\mathbf{F}(t) \\ \mathbf{D}\cdot\mathbf{F}(t) & \mathbf{r}\cdot\mathbf{F}(t) \end{pmatrix}\Psi(t)\,. \tag{6}$$

Here the ion Hamiltonian can be also written as

$$H_{\text{ion}} = \begin{pmatrix} \mathcal{E}_B & 0 \\ 0 & \mathcal{E}_C \end{pmatrix}, \tag{7}$$

h is the one-electron Coulomb Hamiltonian, $\mathbf{F}(t) = \mathbf{F}_0\cos\omega t$ is the IR field strength, $\boldsymbol{d}$ is the transition dipole moment between the B and C residual ion states, and $\mathbf{r}$ is the one-electron operator of dipole moment in the length gauge.

Time-dependent amplitudes

We want to evaluate transition amplitudes, which are later used for calculation of RABBIT signal. If the time-dependent wavefunction $\Psi(t)$ of the system is known and we are interested

in transitions (projections) into the detector state $\Psi_f(t)$, we get a generally time-dependent amplitude as

$$T(t) = \langle \Psi_f(t) | \Psi(t) \rangle \,. \tag{8}$$

This can be also represented as a time integral of a time derivative,

$$T(t) = \int \frac{\mathrm{d}}{\mathrm{d}t} \langle \Psi_f(t) | \Psi(t) \rangle \mathrm{d}t \,. \tag{9}$$

The final state is defined in the field-free setting, so it satisfies the equation $\mathrm{i}\dot{\Psi}_f(t) = [H_{\text{ion}} + h]\Psi_f(t)$. This allows us to differentiate the product and simplify,

$$\begin{aligned} T(t) &= \int \left[\langle \frac{\mathrm{d}}{\mathrm{d}t} \Psi_f(t) | \Psi(t) \rangle + \langle \Psi_f(t) | \frac{\mathrm{d}}{\mathrm{d}t} \Psi(t) \rangle \right] \mathrm{d}t \\ &= \int \left[\langle -\mathrm{i}[H_{\text{ion}} + h] \Psi_f(t) | \Psi(t) \rangle - \mathrm{i} \langle \Psi_f(t) | \mathrm{i} \frac{\mathrm{d}}{\mathrm{d}t} | \Psi(t) \rangle \right] \mathrm{d}t \\ &= -\mathrm{i} \int \langle \Psi_f(t) | \left[\mathrm{i} \frac{\mathrm{d}}{\mathrm{d}t} - H_{\text{ion}} - h \right] | \Psi(t) \rangle \mathrm{d}t \,. \end{aligned} \tag{10}$$

Specifically, we will be using the formula

$$T = -\mathrm{i} \int_{-\infty}^{\infty} \langle \Psi_f(t) | \left[\mathrm{i} \frac{\mathrm{d}}{\mathrm{d}t} - H_{\text{ion}} - h \right] | \Psi(t) \rangle \mathrm{d}t \,. \tag{11}$$

Time-dependent perturbation theory

Let us begin lightly and solve Eq. (6) within the time-dependent perturbation theory, taking advantage of the (assumed) fact that the IR field is fairly weak, $|\mathbf{F}_0| \ll 1$. This amounts to reorganizing the sides to

$$\left[\mathrm{i} \frac{\mathrm{d}}{\mathrm{d}t} - H_{\text{ion}} - h \right] \Psi(t) = - \begin{pmatrix} \mathbf{r} \cdot \mathbf{F}(t) & \mathbf{D} \cdot \mathbf{F}(t) \\ \mathbf{D} \cdot \mathbf{F}(t) & \mathbf{r} \cdot \mathbf{F}(t) \end{pmatrix} \Psi(t) \tag{12}$$

and using the zeroth-order, "intermediate" solution (4) on the right-hand side. The left-hand side will then provide the next order, in our case the two-photon wavefunction. Interestingly, the left-hand side is already prepared for the extraction of the amplitude, Eq. (11). We are

interested in ionisation into a specific final state, so we use as the final wavefunction $\Psi_f(t)$ one of the following two options:

$$\Psi_{Bf}(t) = \psi_{Bf}^{(-)} \mathrm{e}^{-\mathrm{i}e_{Bf}t} \Phi_B \mathrm{e}^{-\mathrm{i}\mathcal{E}_B t}\,, \qquad \text{or} \qquad \Psi_{Cf}(t) = \psi_{Cf}^{(-)} \mathrm{e}^{-\mathrm{i}e_{Cf}t} \Phi_C \mathrm{e}^{-\mathrm{i}\mathcal{E}_C t}\,. \tag{13}$$

Projection on $\Psi_{Bf}(t)$ leads to

$$\begin{aligned} T_B = \mathrm{i} \int_{-\infty}^{\infty} \Big[&\langle \psi_{Bf}^{(-)} | \mathbf{r} \cdot \mathbf{F}_0 | \psi_{Bn}^{(+)} \rangle \cos\omega t \, \mathrm{e}^{\mathrm{i}(e_{Bf} - e_{Bn})t} \\ &+ \langle \psi_{Bf}^{(-)} | \psi_{Cn}^{(+)} \rangle \mathbf{D} \cdot \mathbf{F}_0 \cos\omega t \, \mathrm{e}^{\mathrm{i}(\mathcal{E}_B + e_{Bf} - \mathcal{E}_C - e_{Cn})t} \Big] \, \mathrm{d}t\,. \end{aligned} \tag{14}$$

We can pick one or the other complex exponential forming $\cos\omega t = (\mathrm{e}^{\mathrm{i}\omega t} + \mathrm{e}^{-\mathrm{i}\omega t})/2$, corresponding either to emission or absorption of an IR quantum. Performing the time integration then leads to

$$\begin{aligned} T_B = {}& 2\pi\mathrm{i}\delta(e_{Bf} - e_{Bn} \pm \omega) \langle \psi_{Bf}^{(-)} | \mathbf{r} \cdot \tfrac{1}{2}\mathbf{F}_0 | \psi_{Bn}^{(+)} \rangle \\ &+ 2\pi\mathrm{i}\delta(\mathcal{E}_B + e_{Bf} - \mathcal{E}_C - e_{Cn} \pm \omega) \langle \psi_{Bf}^{(-)} | \psi_{Cn}^{(+)} \rangle \mathbf{D} \cdot \tfrac{1}{2}\mathbf{F}_0\,. \end{aligned} \tag{15}$$

Similarly, when projecting on Ψ_{Cf},

$$\begin{aligned} T_C = {}& 2\pi\mathrm{i}\delta(e_{Cf} - e_{Cn} \pm \omega) \langle \psi_{Cf}^{(-)} | \mathbf{r} \cdot \tfrac{1}{2}\mathbf{F}_0 | \psi_{Cn}^{(+)} \rangle \\ &+ 2\pi\mathrm{i}\delta(\mathcal{E}_C + e_{Cf} - \mathcal{E}_B - e_{Bn} \pm \omega) \langle \psi_{Cf}^{(-)} | \psi_{Bn}^{(+)} \rangle \mathbf{D} \cdot \tfrac{1}{2}\mathbf{F}_0\,. \end{aligned} \tag{16}$$

These are well-known asymptotic formulas. The first term corresponds to the "continuum-continuum" transition, the second to the "ion-ion" transition. However, they are rather opaque regarding the physical origin of the "ion-ion" contribution.

Driven two-level system dynamics

In reality, the IR field significantly affects the residual ion. The wavefunction of the ion will evolve according to the two-level time-dependent Schrödinger equation

$$\mathrm{i}\frac{\mathrm{d}}{\mathrm{d}t}\Phi(t) = H_{\text{ion}}\Phi(t) - \begin{pmatrix} 0 & \mathbf{D} \cdot \mathbf{F}(t) \\ \mathbf{D} \cdot \mathbf{F}(t) & 0 \end{pmatrix} \Phi(t)\,. \tag{17}$$

As before, we will operate in the perturbation regime, $|\mathbf{F}_0| \ll 1$ and use the corresponding $\Phi^{(0)}(t)$ everywhere in product with $\mathbf{F}(t)$. Additionally, we structure the unknown solution as

$$\Phi_B(t) = \begin{pmatrix} \alpha_B(t)\mathrm{e}^{-\mathrm{i}\mathcal{E}_B t} \\ \beta_B(t)\mathrm{e}^{-\mathrm{i}\mathcal{E}_C t} \end{pmatrix} \tag{18}$$

and similarly for $\Phi_C(t)$. We get

$$\left[\mathrm{i}\frac{\mathrm{d}}{\mathrm{d}t} - H_{\text{ion}}\right]\Phi_B(t) = \mathrm{i}\begin{pmatrix} \dot{\alpha}_B(t)\mathrm{e}^{-\mathrm{i}\mathcal{E}_B t} \\ \dot{\beta}_B(t)\mathrm{e}^{-\mathrm{i}\mathcal{E}_C t} \end{pmatrix} = -\begin{pmatrix} 0 \\ \mathbf{D}\cdot\mathbf{F}(t)\mathrm{e}^{-\mathrm{i}\mathcal{E}_B t} \end{pmatrix}, \tag{19}$$

leading to

$$\alpha_B(t) = 1\,, \qquad \beta_B(t) = \mathrm{i}\mathbf{D}\cdot\tfrac{1}{2}\mathbf{F}_0\int \mathrm{e}^{-\mathrm{i}\delta t}\mathrm{d}t\,. \tag{20}$$

In the last equation we performed "rotating wave approximation" by keeping only one of the complex exponentials forming the cosine (the one that results in a flatter phase factor) and defined the "detuning" $\delta = \omega - \omega_0$, where $\omega_0 = \mathcal{E}_C - \mathcal{E}_B$. A similar analysis can be done for time evolution of the C state as well. Altogether we get

$$\Phi_B(t) = \begin{pmatrix} \mathrm{e}^{-\mathrm{i}\mathcal{E}_B t} \\ \mathrm{i}\mathrm{e}^{-\mathrm{i}\mathcal{E}_C t}\mathbf{D}\cdot\tfrac{1}{2}\mathbf{F}_0\int \mathrm{e}^{-\mathrm{i}\delta t}\mathrm{d}t \end{pmatrix}, \qquad \Phi_C(t) = \begin{pmatrix} \mathrm{i}\mathrm{e}^{-\mathrm{i}\mathcal{E}_B t}\mathbf{D}\cdot\tfrac{1}{2}\mathbf{F}_0\int \mathrm{e}^{\mathrm{i}\delta t}\mathrm{d}t \\ \mathrm{e}^{-\mathrm{i}\mathcal{E}_C t} \end{pmatrix}. \tag{21}$$

Note that while here we resorted to perturbation treatment for simplicity, one can obtain non-perturbative solutions as well, though still limited by the rotating wave approximation. These are

$$\Phi_B^{NP}(t) = \mathrm{e}^{-\mathrm{i}\lambda t/2}\begin{pmatrix} \mathrm{e}^{\mathrm{i}\delta t/2}\cos\frac{\theta}{2}\mathrm{e}^{-\mathrm{i}\mathcal{E}_B t} \\ -\mathrm{e}^{-\mathrm{i}\delta t/2}\sin\frac{\theta}{2}\mathrm{e}^{-\mathrm{i}\mathcal{E}_C t} \end{pmatrix}, \qquad \Phi_C^{NP}(t) = \mathrm{e}^{+\mathrm{i}\lambda t/2}\begin{pmatrix} \mathrm{e}^{\mathrm{i}\delta t/2}\sin\frac{\theta}{2}\mathrm{e}^{-\mathrm{i}\mathcal{E}_B t} \\ \mathrm{e}^{-\mathrm{i}\delta t/2}\cos\frac{\theta}{2}\mathrm{e}^{-\mathrm{i}\mathcal{E}_C t} \end{pmatrix}, \tag{22}$$

where $\lambda = \sqrt{D^2+\delta^2}$, $D = \mathbf{D}\cdot\mathbf{F}_0$, $\sin\theta = D/\lambda$ and $\cos\theta = \delta/\lambda$. It is an easy exercise to verify that (22) reduces to (21) for $|\mathbf{F}_0| \to 0$. In the following we will be using only the perturbative results (21).

Photoelectron coupled to driven two-level system

We now combine the previous considerations. When the IR field is on, the total time-dependent wave-function of the system is

$$\Psi(t) = \psi_B(t)\Phi_B(t) + \psi_C(t)\Phi_C(t)\,. \tag{23}$$

In presence of dipole coupling, the residual ion states $\Phi_B(t)$ and $\Phi_C(t)$ are no longer plain stationary states, but have to solve the coupled equation

$$\mathrm{i}\frac{\mathrm{d}}{\mathrm{d}t}\Phi(t) = [H_{\text{ion}} - \mathbf{D}\cdot\mathbf{F}(t)\sigma_x]\Phi(t)\,. \tag{24}$$

This implies that the full time-dependent Schrödinger equation

$$\mathrm{i}\frac{\mathrm{d}}{\mathrm{d}t}\Psi(t) = [h + H_{\text{ion}} - \mathbf{r}\cdot\mathbf{F}(t) - \mathbf{D}\cdot\mathbf{F}(t)\sigma_x]\Psi(t)\,, \tag{25}$$

when projected on $\Phi_B(t)$ and $\Phi_C(t)$ reduces to

$$\mathrm{i}\frac{\mathrm{d}}{\mathrm{d}t}\psi_B(t) = [h - \mathbf{r}\cdot\mathbf{F}(t)]\psi_B(t)\,, \tag{26}$$

$$\mathrm{i}\frac{\mathrm{d}}{\mathrm{d}t}\psi_C(t) = [h - \mathbf{r}\cdot\mathbf{F}(t)]\psi_C(t)\,, \tag{27}$$

respectively.[1] The coupling is gone! The photoelectron is not affected by the residual ion dynamics anymore. It is just that $\Phi_C(t)$ contains a small component proportional to Φ_B. As a result, the wavefunction associated with the field-free B channel actually mixes XUV ionisation signal from the original B and C channels, resulting in a perceived *coupling* delay. This is demonstrated in the next subsection. Here we conclude with the solution of the one-electron systems in time-dependent perturbation theory. Actually, simply reorganizing the terms is

[1] We assume orthogonality between $\Phi_B(t)$ and $\Phi_C(t)$ at all times. It can be shown that both (22) and (21) describe a pair of states that are orthogonal at all times.

enough for the later use:

$$\left[\mathrm{i}\frac{\mathrm{d}}{\mathrm{d}t} - h\right]\psi_B(t) = -\mathbf{r}\cdot\mathbf{F}(t)\psi_{Bn}^{(+)}\mathrm{e}^{-\mathrm{i}e_{Bn}t}\,, \tag{28}$$

$$\left[\mathrm{i}\frac{\mathrm{d}}{\mathrm{d}t} - h\right]\psi_C(t) = -\mathbf{r}\cdot\mathbf{F}(t)\psi_{Cn}^{(+)}\mathrm{e}^{-\mathrm{i}e_{Cn}t}\,. \tag{29}$$

RABBIT delays

We now know the complete wavefunction of the system (23), where the time-dependent residual ion states are given within the rotating phase approximation and to the first order in field strength by Eq. (21), and the photoelectron wavefunctions are given to the first order in field strength by Eqs. (28) and (29). The amplitude of detecting the residual ion in a given state and the photoelectron in the given momentum state can be obtained from (11). We first calculate the action of the operator to the right:

$$\left[\mathrm{i}\frac{\mathrm{d}}{\mathrm{d}t} - H_{\mathrm{ion}} - h\right]\Psi(t) = \begin{pmatrix} \mathrm{e}^{-\mathrm{i}\mathcal{E}_B t}[(\mathrm{i}\frac{\mathrm{d}}{\mathrm{d}t} - h)\psi_B(t) - \frac{1}{2}\mathbf{D}\cdot\mathbf{F}_0\psi_{Cn}^{(+)}\mathrm{e}^{\mathrm{i}(\omega-\mathcal{E}_C+\mathcal{E}_B-e_{Cn})t}] \\ \mathrm{e}^{-\mathrm{i}\mathcal{E}_C t}[(\mathrm{i}\frac{\mathrm{d}}{\mathrm{d}t} - h)\psi_C(t) - \frac{1}{2}\mathbf{D}\cdot\mathbf{F}_0\psi_{Bn}^{(+)}\mathrm{e}^{-\mathrm{i}(\omega-\mathcal{E}_C+\mathcal{E}_B+e_{Bn})t}] \end{pmatrix} \tag{30}$$

Now we project on the two possible classes of final states (13),

$$\Psi_{Bf}(t) = \begin{pmatrix} \psi_{Bf}^{(-)}\mathrm{e}^{-\mathrm{i}(e_{Bf}+\mathcal{E}_B)t} \\ 0 \end{pmatrix}\,, \qquad \Psi_{Cf}(t) = \begin{pmatrix} 0 \\ \psi_{Cf}^{(-)}\mathrm{e}^{-\mathrm{i}(e_{Cf}+\mathcal{E}_C)t} \end{pmatrix} \tag{31}$$

and integrate over time. We obtain identical formulas to those in Eqs. (15) and (16)! This confirms that the picture with the residual ion freely Rabi-cycling in the IR field gives consistent results with the straightforward application of the time-dependent and time-independent perturbation theory to the coupled system. However, it gives better physical insight, as it actually isolates the ion-ion transition fully from the photoelectron dynamics. Instead, the ion-ion ("coupling") RABBIT delay contribution arises in mixing of one-electron wave functions originally coupled to a given pair of states that is later rotated in the $B-C$ space by the IR-driven coupling.

Sign of coupling delay

The coupling delay arises in the interference of pathway cross-terms

$$\tau_{\text{coupl}} \simeq \frac{1}{2\omega}\mathrm{Im}\frac{\delta Q}{Q}\,, \tag{32}$$

where

$$\delta Q = T^{(2)*}_{+,\text{free-free}} T^{(2)}_{-,\text{ion-ion}} + T^{(2)*}_{+,\text{ion-ion}} T^{(2)}_{-,\text{free-free}}\,. \tag{33}$$

In the asymptotic approximation, the intermediate state in the XUV±IR pathways can be represented using the one-photon matrix element and the outgoing Coulomb function with intermediate momentum $\kappa_\pm$, while the final wave function is proportional to the standing-wave Coulomb function with final momentum k,

$$\psi^{(+)}_{n\pm} \sim \frac{1}{r}\sum_{lm} \mathrm{i}^l \mathrm{e}^{-\mathrm{i}\sigma_l(\kappa_\pm)} d^{(1)}_{\pm,lm} H^+_l(\kappa_\pm, r) Y_{lm}(\mathbf{r})\,, \tag{34}$$

$$\psi^{(-)}_{f} \sim \frac{1}{r}\sum_{lm} \mathrm{i}^l \mathrm{e}^{-\mathrm{i}\sigma_l(k)} F_l(k, r) Y_{lm}(\mathbf{r}) Y^*_{lm}(\boldsymbol{k})\,. \tag{35}$$

As a result, the ion-ion contribution to the two-photon matrix element can be written

$$T^{(2)}_{\pm,\text{ion-ion}} \sim \mathbf{D}\cdot\tfrac{1}{2}\mathbf{F}_0 \langle \psi^{(-)}_f | \psi^{(+)}_{n\pm}\rangle \sim \mathbf{D}\cdot\tfrac{1}{2}\mathbf{F}_0 \sum_{lm} d^{(1)}_{\pm,lm} A^{(0)}_{\kappa lkl}\,. \tag{36}$$

The radial integral of two Coulomb functions has a closed form,

$$\begin{aligned} A^{(0)}_{\kappa lkl} &= -\frac{2}{\sqrt{\kappa k}} \mathrm{e}^{\mathrm{i}\sigma_l(k)-\mathrm{i}\sigma_l(\kappa)} \int_0^{+\infty} F_l(k,r) H^+_l(\kappa_\pm, r)\mathrm{d}r \\ &= \frac{2}{k^2-\kappa_\pm^2} \frac{k^{l+1/2} e^{Z\pi/2k} |\Gamma(l+1-Zi/k)|}{\kappa_\pm^{l+1/2} e^{Z\pi/2\kappa} |\Gamma(l+1-Zi/\kappa_\pm)|} \\ &\to \frac{2}{k^2-\kappa_\pm^2} = \frac{-1}{\Delta_{fn}\mp\omega}\,. \qquad [k\to\infty] \end{aligned} \tag{37}$$

This way, $T^{(2)}_{+,\text{ion-ion}}$ and $T^{(2)}_{-,\text{ion-ion}}$ have quite different magnitudes depending on the relation between IR energy ω and the difference between the intermediate and the final state energies

$\Delta_{fn} = \mathcal{E}_f - \mathcal{E}_n$. Close to the resonant transition ($\omega \sim \Delta_{fn}$) one of these two amplitudes is dominant, leaving only one of the two terms in (33). This dominant ion-ion amplitude, proportional to $1/(|\omega| - |\Delta_{fn}|)$, also controls the overall sign of the whole expression. In other words, the sign-dependence arises in the integral (37). The integral's sign simply depends on whether the intermediate or the final kinetic energy of the photoelectron is larger.

We illustrate sign dependence of coupling delay below with our large multichannel channel calculation of RABBIT in CO_2, see Supplementary Fig. 9. When the non-zero transition dipole is explicitly removed from the calculation, the resulting RABBIT delays for IR wavelengths 800 nm and 1030 nm are quite similar at higher energies. When the $B-C$ coupling is included, the two curves separate into opposite directions. The solid and dash-dot curves present results of the proper two-photon (PT-2) calculation, while the dotted curves come from the asymptotic approximation $\tau_R \approx \tau_1 + \tau_{\rm coupl} + \tau_{cc}$. The figure suggests that above 40 eV the latter separability holds very well.

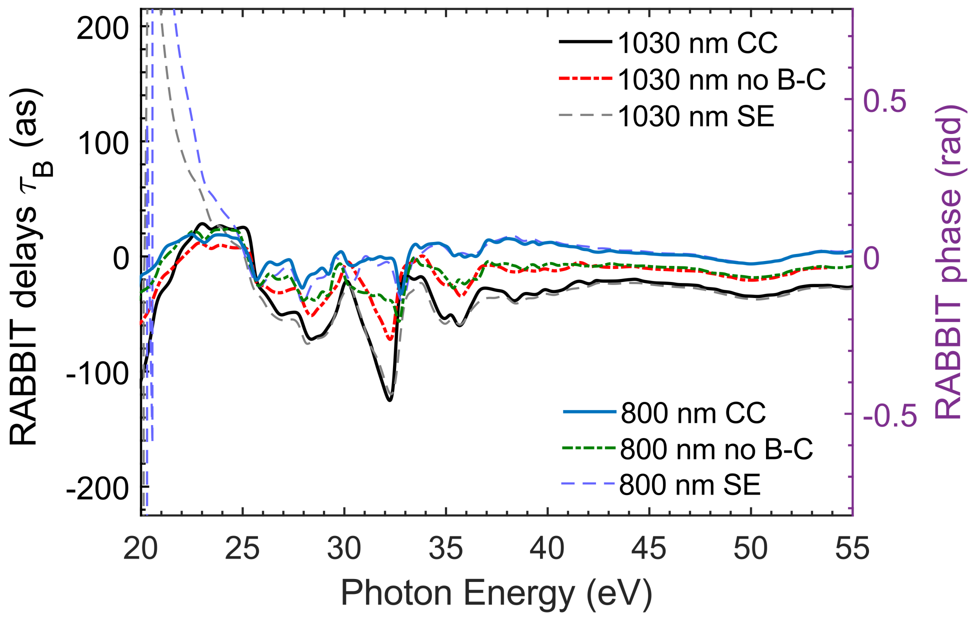

Supplementary Fig. 9: **Coupling delay for different wavelengths of the driving field.** Coupling delay with 800 and 1030 nm in $B\,^2\Sigma_u$ of $CO_2{}^+$, with and without the inclusion of the transition dipole. All curves present delays obtained from the smoothed amplitudes. Some datasets haven't been calculated all the way to the right edge of the plot.

Reduced density matrix

The total time-dependent density operator is

$$\hat{\rho}_{\text{tot}}(t) = |\Psi(t)\rangle\langle\Psi(t)|\,, \tag{38}$$

where the state $\Psi(t)$ is assumed in the high-energy high-distance form

$$\Psi(t) = \psi_B(t)\Phi_B(t) + \psi_C(t)\Phi_C(t)\,. \tag{39}$$

We will use the momentum basis $\{|\boldsymbol{k}\rangle\}$ for the photoelectron wavefunction, assuming that it is complete,

$$\int |\boldsymbol{k}\rangle\langle\boldsymbol{k}|\mathrm{d}^3\boldsymbol{k} = 1\,. \tag{40}$$

We define the reduced density matrix for the ion subsystem as

$$\hat{\rho}_{\text{ion}}(t) = \int \langle \boldsymbol{k} | \hat{\rho}_{\text{tot}}(t) | \boldsymbol{k} \rangle \mathrm{d}^3 \boldsymbol{k} \, . \tag{41}$$

Substituting from Eq. (39), this can be expanded to

$$\begin{aligned} \hat{\rho}_{\text{ion}}(t) = {} & |\Phi_B(t)\rangle\langle\Phi_B(t)| \int \langle\psi_B(t)|\boldsymbol{k}\rangle\langle\boldsymbol{k}|\psi_B(t)\rangle \mathrm{d}^3\boldsymbol{k} + |\Phi_B(t)\rangle\langle\Phi_C(t)| \int \langle\psi_C(t)|\boldsymbol{k}\rangle\langle\boldsymbol{k}|\psi_B(t)\rangle \mathrm{d}^3\boldsymbol{k} \\ & + |\Phi_C(t)\rangle\langle\Phi_B(t)| \int \langle\psi_B(t)|\boldsymbol{k}\rangle\langle\boldsymbol{k}|\psi_C(t)\rangle \mathrm{d}^3\boldsymbol{k} + |\Phi_C(t)\rangle\langle\Phi_C(t)| \int \langle\psi_C(t)|\boldsymbol{k}\rangle\langle\boldsymbol{k}|\psi_C(t)\rangle \mathrm{d}^3\boldsymbol{k} \, . \end{aligned} \tag{42}$$

Now, taking advantage of the assumed completeness of our momentum basis, Eq. (40), this simplifies to

$$\begin{aligned} \hat{\rho}_{\text{ion}}(t) = {} & |\Phi_B(t)\rangle\langle\Phi_B(t)| \cdot ||\psi_B(t)||^2 + |\Phi_B(t)\rangle\langle\Phi_C(t)| \cdot \langle\psi_C(t)|\psi_B(t)\rangle \\ & + |\Phi_C(t)\rangle\langle\Phi_B(t)| \cdot \langle\psi_B(t)|\psi_C(t)\rangle + |\Phi_C(t)\rangle\langle\Phi_C(t)| \cdot ||\psi_C(t)||^2 \, , \end{aligned} \tag{43}$$

or in two-level space with basis $\{\Phi_B(t), \Phi_C(t)\}$ just

$$\rho_{\text{ion}} = \begin{pmatrix} ||\psi_B(t)||^2 & \langle\psi_B(t)|\psi_C(t)\rangle^* \\ \langle\psi_B(t)|\psi_C(t)\rangle & ||\psi_C(t)||^2 \end{pmatrix} = \begin{pmatrix} ||\psi_B||^2 & \langle\psi_B|\psi_C\rangle^* \\ \langle\psi_B|\psi_C\rangle & ||\psi_C||^2 \end{pmatrix} . \tag{44}$$

The last equality holds because the time evolution is unitary, so all inner products remain the same at all times. This follows from the fact that $\psi_B(t)$ and $\psi_C(t)$ satisfy decoupled equations and each can be written as $\psi_X(t) = \exp[-\mathrm{i}\hat{H}t/\hbar]\psi_X$, where the Hamiltonian $\hat{H}$ is the same for both states. The off-diagonal terms of ρ_{ion} vanish if the photoelectron wavefunctions in the two channels are orthogonal (e.g.) right after the XUV ionization. This seems to be the case, as the two stationary states have necessarily different symmetries (Σ_g^+ vs. Σ_u^+). This follows from parity conservation: The initial neutral state has symmetry Σ_g^+, the state after absorption of an XUV photon polarized along z has symmetry Σ_u^+, so the photoelectron wavefunctions have to complement the residual ion symmetries Σ_u^+ (B state) and Σ_g^+ (C state) within this latter total wavefunction symmetry. As a result,

$$\rho_{\text{ion}} = \begin{pmatrix} ||\psi_B||^2 & 0 \\ 0 & ||\psi_C||^2 \end{pmatrix} , \tag{45}$$

where the diagonal elements are essentially the XUV cross sections for the two channels. This is how the reduced density matrix looks like in the time-dependent (dressed) basis $\{|\Phi_B(t)\rangle, |\Phi_C(t)\rangle\}$. To transform it to the basis of the field-free states, $\{|\Phi_B\rangle, |\Phi_C\rangle\}$, we need to change basis. These two bases are connected by a unitary transformation $U(t)$,

$$\begin{pmatrix} |\Phi_B(t)\rangle \\ |\Phi_C(t)\rangle \end{pmatrix} = U(t) \begin{pmatrix} |\Phi_B\rangle \\ |\Phi_C\rangle \end{pmatrix} = \begin{pmatrix} u_{BB} & u_{BC} \\ u_{CB} & u_{CC} \end{pmatrix} \begin{pmatrix} |\Phi_B\rangle \\ |\Phi_C\rangle \end{pmatrix}, \tag{46}$$

representing the driven two-level system dynamics. The basis representation of Eq. (45) can be written in the abstract way as

$$\hat{\rho}_{\text{ion}} = \begin{pmatrix} |\Phi_B(t)\rangle & |\Phi_C(t)\rangle \end{pmatrix} \cdot \begin{pmatrix} ||\psi_B||^2 & 0 \\ 0 & ||\psi_C||^2 \end{pmatrix} \cdot \begin{pmatrix} \langle\Phi_B(t)| \\ \langle\Phi_C(t)| \end{pmatrix}, \tag{47}$$

which is equivalent to

$$\hat{\rho}_{\text{ion}} = \begin{pmatrix} |\Phi_B\rangle & |\Phi_C\rangle \end{pmatrix} \cdot U(t)^{\top} \cdot \begin{pmatrix} ||\psi_B||^2 & 0 \\ 0 & ||\psi_C||^2 \end{pmatrix} \cdot U(t)^{*} \cdot \begin{pmatrix} \langle\Phi_B| \\ \langle\Phi_C| \end{pmatrix}. \tag{48}$$

We see that the density matrix in this basis generally has a dense structure; denoting $b_+ = ||\psi_B||^2$ and $c_+ = ||\psi_C||^2$ we get

$$\hat{\rho}_{\text{ion}} = \begin{pmatrix} |\Phi_B\rangle & |\Phi_C\rangle \end{pmatrix} \cdot \begin{pmatrix} b_+|u_{BB}|^2 + c_+|u_{CB}|^2 & b_+u_{BB}u_{BC}^* + c_+u_{CB}u_{CC}^* \\ b_+u_{BB}^*u_{BC} + c_+u_{CB}^*u_{CC} & b_+|u_{BC}|^2 + c_+|u_{CC}|^2 \end{pmatrix} \cdot \begin{pmatrix} \langle\Phi_B| \\ \langle\Phi_C| \end{pmatrix}. \tag{49}$$

Trace of the matrix in the basis $\{\Phi_B, \Phi_C\}$ is

$$\operatorname{Tr} \hat{\rho}_{\text{ion}} = b_+ \underbrace{\left(|u_{BB}|^2 + |u_{BC}|^2\right)}_{1} + c_+ \underbrace{\left(|u_{CB}|^2 + |u_{CC}|^2\right)}_{1} = b_+ + c_+ \tag{50}$$

due to unit norm of rows and columns in the unitary matrix U. This agrees with the trace of the reduced density matrix in the basis $\{\Phi_B(t), \Phi_C(t)\}$, Eq. (45). If $b_+ = c_+$, the off-diagonal elements in Eq. (49) will evaluate to zero due to orthogonality of rows and columns of U. However, for a general case, the off-diagonal elements *are* present when the reduced density matrix is represented in the time-independent residual ion state basis.

Purity and coupling delay

Following the previous section we normalize the reduced density matrix so that it has unit trace,

$$\rho_{\text{ion}} = \frac{1}{b_+ + c_+} \begin{pmatrix} b_+ & 0 \\ 0 & c_+ \end{pmatrix} . \tag{51}$$

Trace of square of this matrix, the purity, is then

$$\gamma = \mathrm{Tr}\, \rho_{\text{ion}}^2 = \frac{b_+^2 + c_+^2}{(b_+ + c_+)^2} . \tag{52}$$

From this we can express the ratio c_+/b_+ as

$$\frac{c_+}{b_+} = \frac{\gamma - \sqrt{2\gamma - 1}}{1 - \gamma} . \tag{53}$$

where we assumed $c_+ < b_+$ when choosing one of the two solutions of the corresponding quadratic equation. RABBIT delay in a three-path RABBIT into the $B^2\Sigma_u^+$ state can be obtained from the two-photon ionization signal

$$I \sim \left| T_{BB,+}^{(2)} + \left(T_{BB,-}^{(2)} + T_{CB,-}^{(2)} \right) \right|^2 . \tag{54}$$

Here $T_{BB,\pm}^{(2)}$ denotes the two-photon ionization amplitude into the $B^2\Sigma_u^+$ state that involves absorption or emission of the IR photon by the photoelectron, while $T_{CB,-}^{(2)}$ is the two-photon amplitude into $B^2\Sigma_u^+$ state via the $C^2\Sigma_g^+$ state, involving emission of IR photon in the residual ion. The oscillating RABBIT interference term is then

$$Q = T_{BB,+}^{(2)*} \left(T_{BB,-}^{(2)} + T_{CB,-}^{(2)} \right) . \tag{55}$$

If emission direction and/or molecular orientation is not resolved, we need to integrate Q over the corresponding angles. This will be denoted by the angle brackets as

$$\langle Q \rangle = \left\langle T_{BB,+}^{(2)*} \left(T_{BB,-}^{(2)} + T_{CB,-}^{(2)} \right) \right\rangle . \tag{56}$$

The complete delay is

$$\tau_R = \frac{1}{2\omega} \arg \left\langle T^{(2)*}_{BB,+} \left(T^{(2)}_{BB,-} + T^{(2)}_{CB,-} \right) \right\rangle . \tag{57}$$

This can be split as

$$\tau_R = \frac{1}{2\omega} \arg \left\langle T^{(2)*}_{BB,+} T^{(2)}_{BB,-} \right\rangle + \frac{1}{2\omega} \arg \left(1 + \frac{\left\langle T^{(2)*}_{BB,+} T^{(2)}_{CB,-} \right\rangle}{\left\langle T^{(2)*}_{BB,+} T^{(2)}_{BB,-} \right\rangle} \right) . \tag{58}$$

The first term is the standard two-pathway RABBIT and can be written at high energies aproximately as $\tau_W + \tau_{cc}$. The second term is the coupling delay τ_{ii} (indicated as τ_{ion} in the main manuscript), so we can write

$$\tau_R \sim \tau_W + \tau_{cc} + \tau_{ii} \, . \tag{59}$$

At high energies we may approximate the amplitudes using asymptotic formulas[2],

$$T^{(2)}_{BB,\pm} \sim \sum_{lm\lambda\mu} Y_{lm} A^{(1)}_{\kappa_\pm \lambda k l} \langle lm | \hat{\boldsymbol{n}} \cdot \hat{\boldsymbol{\epsilon}}_{\mathrm{IR}} | \lambda\mu \rangle d^{(1)}_{B,\lambda\mu}(\kappa_\pm) \, , \tag{60}$$

$$T^{(2)}_{CB,-} \sim (\mathbf{D}_{BC} \cdot \hat{\boldsymbol{\epsilon}}_{\mathrm{IR}}) \sum_{lm} Y_{lm} A^{(0)}_{\kappa_C lkl} d^{(1)}_{C,lm}(\kappa_C) \, , \tag{61}$$

where $d^{(1)}_{nlm}$ are the partial-wave resolved one-photon ionization amplitudes at the intermediate energies (after XUV absorption). The radial integrals $A^{(w)}_{\kappa\lambda kl}$ have the high-energy limits[2]

$$A^{(0)}_{\kappa lkl} \sim \frac{-1}{\Delta - \omega_{\mathrm{IR}}} \, , \tag{62}$$

$$A^{(1)}_{\kappa\lambda kl} \sim \frac{\mathrm{i}k}{\omega^2_{\mathrm{IR}}} \, , \tag{63}$$

where $\Delta = \mathcal{E}_C - \mathcal{E}_B$ is the energy difference between the $B^2\Sigma^+_u$ and $C^2\Sigma^+_g$ states. So,

$$T^{(2)}_{BB,\pm} \sim \frac{\mathrm{i}k}{\omega^2_{\mathrm{IR}}} \sum_{lm\lambda\mu} Y_{lm} \langle lm | \hat{\boldsymbol{n}} \cdot \hat{\boldsymbol{\epsilon}}_{\mathrm{IR}} | \lambda\mu \rangle d^{(1)}_{B,\lambda\mu}(\kappa_\pm) = \frac{\mathrm{i}k}{\omega^2_{\mathrm{IR}}} (\hat{\boldsymbol{k}} \cdot \hat{\boldsymbol{\epsilon}}_{\mathrm{IR}}) d^{(1)}_B(\kappa_\pm \hat{\boldsymbol{k}}) \, , \tag{64}$$

$$T^{(2)}_{CB,-} \sim \frac{-1}{\Delta - \omega_{\mathrm{IR}}} (\mathbf{D}_{BC} \cdot \hat{\boldsymbol{\epsilon}}_{\mathrm{IR}}) \sum_{lm} Y_{lm} d^{(1)}_{C,lm}(\kappa_C) = \frac{-1}{\Delta - \omega_{\mathrm{IR}}} (\mathbf{D}_{BC} \cdot \hat{\boldsymbol{\epsilon}}_{\mathrm{IR}}) d^{(1)}_C(\kappa_C \hat{\boldsymbol{k}}) \, , \tag{65}$$

The amplitude T_{CB} is generally smaller than T_{BB} by a factor of ω/k and can be thought to be a small perturbation. Additionally, at high energies the one-photon ionization amplitudes do not depend on energy significantly and can be assumed constant in the energy range spanned by the two consecutive XUV harmonics. This allows setting $\kappa_+ \sim \kappa_- \sim \kappa_C$ and simplifying the formula for the coupling delay as

$$\begin{aligned}
\tau_{ii} &\sim \frac{1}{2\omega} \arg \left(1 + \mathrm{i} \frac{\omega_{\mathrm{IR}}^2}{k} \frac{1}{\Delta - \omega_{\mathrm{IR}}} \frac{\langle (\hat{\boldsymbol{k}} \cdot \hat{\boldsymbol{\epsilon}}_{\mathrm{IR}})(\mathbf{D}_{BC} \cdot \hat{\boldsymbol{\epsilon}}_{\mathrm{IR}}) d_B^{(1)*} d_C^{(1)} \rangle}{\langle (\hat{\boldsymbol{k}} \cdot \hat{\boldsymbol{\epsilon}}_{\mathrm{IR}})^2 d_B^{(1)*} d_B^{(1)} \rangle} \right) \\
&\sim \frac{1}{2\omega_{\mathrm{IR}}} \mathrm{Re} \left(\frac{\omega_{\mathrm{IR}}^2}{k} \frac{1}{\Delta - \omega_{\mathrm{IR}}} \frac{\langle (\hat{\boldsymbol{k}} \cdot \hat{\boldsymbol{\epsilon}}_{\mathrm{IR}})(\mathbf{D}_{BC} \cdot \hat{\boldsymbol{\epsilon}}_{\mathrm{IR}}) d_B^{(1)*} d_C^{(1)} \rangle}{\langle (\hat{\boldsymbol{k}} \cdot \hat{\boldsymbol{\epsilon}}_{\mathrm{IR}})^2 d_B^{(1)*} d_B^{(1)} \rangle} \right) \\
&\sim \frac{\omega_{\mathrm{IR}}}{2k} \frac{1}{\Delta - \omega_{\mathrm{IR}}} \frac{\mathrm{Re} \langle (\hat{\boldsymbol{k}} \cdot \hat{\boldsymbol{\epsilon}}_{\mathrm{IR}})(\mathbf{D}_{BC} \cdot \hat{\boldsymbol{\epsilon}}_{\mathrm{IR}}) d_B^{(1)*} d_C^{(1)} \rangle}{\langle (\hat{\boldsymbol{k}} \cdot \hat{\boldsymbol{\epsilon}}_{\mathrm{IR}})^2 |d_B^{(1)}|^2 \rangle}
\end{aligned} \tag{66}$$

If the photoelectron signal is *not* integrated over, the averaging procedure does not affect $\hat{\boldsymbol{k}}$, so it can be pulled out of the angle brackets,

$$\tau_{ii} \sim \frac{1}{2k} \frac{\omega_{\mathrm{IR}}}{\Delta - \omega_{\mathrm{IR}}} \frac{1}{\hat{\boldsymbol{k}} \cdot \hat{\boldsymbol{\epsilon}}_{\mathrm{IR}}} \frac{\mathrm{Re} \langle (\mathbf{D}_{BC} \cdot \hat{\boldsymbol{\epsilon}}_{\mathrm{IR}}) d_B^{(1)*} d_C^{(1)} \rangle}{\langle |d_B^{(1)}|^2 \rangle} \tag{67}$$

Square of $d_B^{(1)}$ is proportional to the one-photon ionization cross section σ_B, typically averaged over orientations of the molecule. Unfortunately, it is difficult to interpret the orientation-averaged expression $\langle (\mathbf{D}_{BC} \cdot \hat{\boldsymbol{\epsilon}}_{\mathrm{IR}}) d_B^* d_C \rangle$. If we instead restrict the analysis to the **molecular frame**, we may simply erase the angle brackets altogether and write the coupling delay using the molecular-frame differential cross sections σ_B, σ_C as

$$\tau_{ii} \sim \frac{1}{2k} \frac{\omega_{\mathrm{IR}}}{\Delta - \omega_{\mathrm{IR}}} \frac{\mathbf{D}_{BC} \cdot \hat{\boldsymbol{\epsilon}}_{\mathrm{IR}}}{\hat{\boldsymbol{k}} \cdot \hat{\boldsymbol{\epsilon}}_{\mathrm{IR}}} \sqrt{\frac{\sigma_C}{\sigma_B}} \cos \arg (d_B^{(1)*} d_C^{(1)}) \,. \tag{68}$$

This formula captures all (asymptotic) features of the coupling delay. Interestingly, the dependence on the cross sections, or on magnitude of the ionization amplitudes, is concentrated to the square root term, which can be written using the earlier-derived purity, yielding

$$\boxed{\tau_{ii} \sim \frac{1}{2k} \frac{\omega_{\mathrm{IR}}}{\Delta - \omega_{\mathrm{IR}}} \frac{\mathbf{D}_{BC} \cdot \hat{\boldsymbol{\epsilon}}_{\mathrm{IR}}}{\hat{\boldsymbol{k}} \cdot \hat{\boldsymbol{\epsilon}}_{\mathrm{IR}}} \sqrt{\frac{\gamma - \sqrt{2\gamma - 1}}{1 - \gamma}} \cos \arg (d_B^{(1)*} d_C^{(1)})} \,. \tag{69}$$

The remaining parameters are the phases of the ionization amplitudes, the detuning of IR from the resonance, the strength of the dipole transition, and projections of the transition dipole and of the photoelectron emission direction along the IR polarization.

The square root factor:

$$X(\gamma) = \sqrt{\frac{\gamma - \sqrt{2\gamma - 1}}{1 - \gamma}} \tag{70}$$

is a monotonically decreasing function of γ, decreasing from 1 at $\gamma = 1/2$ to 0 at $\gamma = 1$, as shown in Supplementary Fig. 10. This means that the function $X(\gamma)$ is positively correlated with the degree of entanglement. As a result, with maximal entanglement we get maximal effect of the coupling delay.

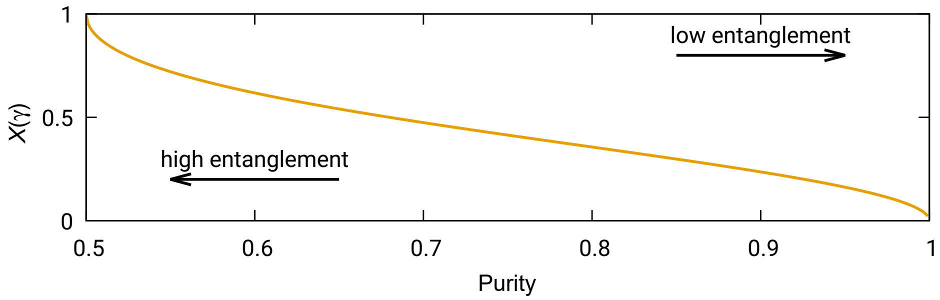

Supplementary Fig. 10: **Effect of entanglement.** Factor $X(\gamma)$ in Eqs. (69) and (70) as a function of the purity γ.

Numerical illustration

RABBIT delay has been calculated in molecular frame for parallel molecular axis, polarization and emission using second-order perturbation theory. This is compared to Wigner delay corrected by continuum-continuum delay (Dahlström LR variant[3]) and by the present ion-ion coupling delay (Eq. (68)). The corresponding datasets are plotted in Supplementary Fig. 11, showing very nice agreement between the first and third approach. The model was based on CASSCF target orbitals with R-matrix radiuss of 10 atomic units, pure B-spline continnum basis (25

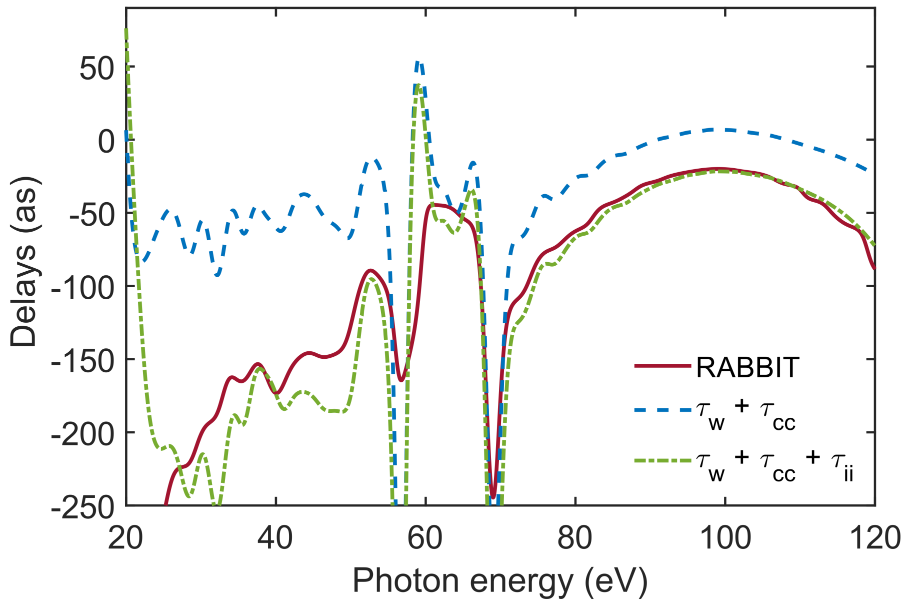

Supplementary Fig. 11: **Ion-ion coupling delay.** Effect of the ion-ion coupling delay (τ_{ii}) in CO_2^+, state $B\,^2\Sigma_u^+$, for 1030-nm IR.

B-splines) and partial wave epxnasion up to $\ell = 9$. The calculation is likely not converged at energies beyond 100 eV with respect to angular momentum expansion. Note that this calculation was performed for $\lambda_{\text{IR}} = 1030$ nm.

The purity of the state calculated according to Eq. (52) is presented in Supplementary Fig. 12.

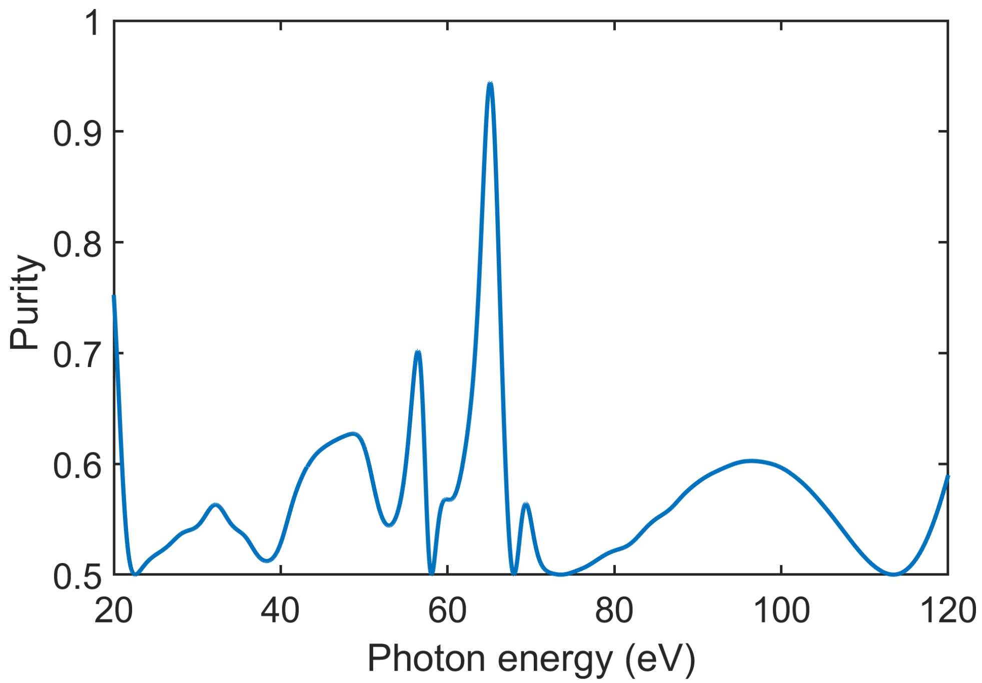

Supplementary Fig. 12: **Purity.** Purity calculated according to Eq. (52).

Supplementary References

[1] Siggel, M. R. F. *et al.* Shape–resonance–enhanced continuum–continuum coupling in photoionization of CO_2. *The Journal of chemical physics* **99**, 1556–1563 (1993).

[2] Benda, J. *et al.* Angular momentum dependence in multiphoton ionization and attosecond time delays. *Physical Review A* **111**, 013110 (2025).

[3] Dahlström, J. *et al.* Theory of attosecond delays in laser-assisted photoionization. *Chemical Physics* **414**, 53–64 (2013).
